# Comparative Analysis of the Tumour Mutational Burden in Erosive and Reticular Oral Lichen Planus

**DOI:** 10.1111/odi.70020

**Published:** 2025-07-04

**Authors:** Priscila Laiza Rubim Leão, Rennan Garcias Moreira, Fernanda Faria Rocha, Laura de Freitas Xavier, Larissa Rany Martins‐Chaves, Ana Carolina Carneiro Batista de Oliveira, Soraya de Mattos Camargo Grossmann Almeida, Silvia Ferreira de Sousa, Marina Gonçalvez Diniz, Roberta Rayra Martins‐Chaves, Ricardo Santiago Gomez

**Affiliations:** ^1^ Department of Oral Surgery and Pathology, School of Dentistry Universidade Federal de Minas Gerais Belo Horizonte Brazil; ^2^ Multiuser Laboratories Center, Center of Genomics, Biological Sciences Institute Universidade Federal de Minas Gerais (UFMG) Belo Horizonte Minas Gerais Brazil; ^3^ Faculty of Medical Sciences of Minas Gerais Belo Horizonte Brazil; ^4^ School of Dentistry Pontifícia Universidade Católica de Minas Gerais Belo Horizonte Brazil; ^5^ Department of Pathology, Biological Sience Institute Universidade Federal de Minas Gerais Belo Horizonte Brazil

**Keywords:** malignant transformation, oral lichen planus, tumour mutation burden

## Abstract

**Objective:**

Oral lichen planus (OLP) is a chronic inflammatory disease classified as an oral potentially malignant lesion. The erosive and reticular forms of OLP have the potential for malignant transformation, with no consistent data indicating that one form is more likely to undergo malignant transformation than the other. Tumour mutational burden (TMB) is a parameter that represents the number of somatic mutations in the DNA of neoplastic cells. This study aimed to compare TMB levels in the two primary clinical forms of OLP, the erosive and reticular sub‐types.

**Methods:**

Next‐generation sequencing of samples from 18 patients with OLP, including nine of each clinical form, was performed using the QIAseq Targeted DNA Human TMB Panel.

**Results:**

Eight (44.4%) of the samples had a TMB ≤ 10 mutations/Mb, while 10 (55.6%) had a TMB of zero. No significant difference was observed between the erosive and reticular forms of OLP. A maximum of two somatic variations per sample was identified, involving genes associated with immune response (*PTPRD*, *PSMA6* and *CD274*) and tumour suppression (*PALB2, ATRX* and *BRCA2*).

**Conclusion:**

Although these data suggest that genetic mutational events occur similarly in erosive and reticular OLP, further molecular studies are required to confirm the findings.

## 1 Introduction

Lichen planus is a chronic inflammatory disease with mucocutaneous involvement that primarily affects females, starting in their third decade of life. It is relatively common, affecting 0.2%–2% of the general population (Torrente‐Castells et al. 2010; Cox et al. 2020). Oral lichen planus (OLP) presents with different clinical manifestations, with the reticular and erosive forms being the most frequently observed in clinical practice. The reticular form is characterised by intertwined, white hyperkeratotic striae that form a lacy pattern known as Wickham striae, predominantly affecting the bilateral buccal mucosa (Cox et al. 2020). Erythematous and atrophic areas characterise the erosive variant and are mostly associated with symptomatic presentations (Cox et al. 2020; Lodi et al. 2020).

The aetiology of OLP remains unclear; however, studies suggest that it is immunologically mediated by a T lymphocyte (cluster of differentiation CD4 and CD8) response, which triggers the release of inflammatory cytokines and promotes the apoptosis of basal keratinocytes (Zhang et al. 2017). The inflammatory process observed in the aetiopathogenesis of OLP is a significant factor in the progression and severity of the clinical condition (Enomoto et al. 2018).

Diagnosis of OLP is based on clinical characteristics in conjunction with histopathological findings. The primary histopathological findings include hyperkeratosis or atrophy, sawtooth‐like epithelial ridges and basal vacuolisation. In the lamina propria, a juxta‐epithelial lymphocytic inflammatory infiltrate is typically observed, mainly composed of T lymphocytes (Cheng et al. 2016).

Periodic monitoring of patients is essential due to the risk of malignant transformation, which has a rate between 0.44% and 2.28% (González‐Moles et al. 2021; Ramos‐García et al. 2021). One study reported that the erosive/ulcerative variant has a 25.8 times greater likelihood of progressing to oral squamous cell carcinoma than the reticular/hypertrophic form (Guan et al. 2020). However, the factors that trigger malignant transformation are poorly understood. Previous studies have noted mutations in genes associated with cell repair and apoptosis, such as *TP53, KMT2D, CASP8* and *FAT1*, in patients with OLP that have undergone malignant transformation (Xie et al. 2022) Additionally, other genetic factors, including the loss of heterozygosity in tumour suppressor genes and aneuploidy (characterised by changes in chromosome number), have been reported in OLP. These factors contribute to genomic instability and uncontrolled cell proliferation (Yahalom et al. 2016).

The number of somatic mutations per megabase (Mb) of interrogated genomic sequence of neoplastic cells is referred to as tumour mutation burden (TMB), which may reflect the potential for tumour progression and oncogenesis in various tumours (Ladányi and Tímár 2020). Some authors have associated a high TMB with increased malignant transformation of gastric metaplasia into intestinal adenocarcinoma (Black et al. 2024), sinonasal papilloma into squamous cell carcinoma (Kwon et al. 2024), and atypical adenomatous hyperplasia into invasive lung adenocarcinoma (Hu et al. 2021). In addition, an increased TMB has been reported in malignantly transformed oral leukoplakia (Hanna et al. 2024). To the best of our knowledge, there are no data on the TMB in OLP. This study aimed to compare the TMB of erosive and reticular variants of OLP.

## 2 Methodology

This cross‐sectional study was approved by the Research Ethics Committee of the Federal University of Minas Gerais (CAAE: 50131721.1.0000.5149) and was carried out under the principles established by the Declaration of Helsinki. Informed consent was obtained from all subjects. Paraffin‐embedded blocks of tissue samples of OLP containing relevant clinical information were selected from the files of the Oral Pathology Laboratory at the Federal University of Minas Gerais and Pontifical Catholic University of Minas Gerais. All the samples were from non‐smokers, and the available clinical information was used to classify the lesions as either reticular or erosive OLP, according to the criteria proposed by the American Academy of Oral and Maxillofacial Pathology (Cheng et al. 2016). After reviewing the clinical information and the histopathological slides, 18 samples containing adequate material for DNA extraction were chosen, including nine samples of each clinical form: erosive or reticular OLP.

Genomic DNA (gDNA) was extracted using the QIAamp DNA FFPE Tissue Kit (Qiagen Inc., Valencia, CA, USA) following the manufacturer's instructions. The gDNA extracted from the 18 samples was subjected to library preparation for subsequent sequencing. The QIAseq Targeted DNA Kit (Qiagen) was used to prepare the libraries, employing a pre‐defined panel for analysing the TMB (QIAseq Targeted DNA Human Tumour Mutational Burden Panel, Qiagen Inc., Valencia, CA, USA). Finally, the samples were sequenced using Illumina technology on a NextSeq 550 system (Fiocruz, Rene Rachou, Belo Horizonte, Brazil) using two NextSeq 550 System Mid‐Output Kit 300‐cycle runs.

Data were analysed using the CLC Genomics Workbench (Qiagen), by the workflow ‘Identify QIAseq DNA Somatic Variants with TMB Score (Illumina)’ available at the plugin ‘Biomedical Genomics Analysis’. To calculate TMB Score, a serie of filtering was applied to remove known germlines variants and variants that are likely to be germline based on the observed variant allele frequency.

To evaluate the clinical significance interpretations of the variants found in the study, we used ClinVar (http://www.ncbi.nlm.nih.gov/clinvar/) database at the National Center for Biotechnology Information (NCBI). ClinVar is a freely accessible, public archive of reports of human genomic variants and interpretations of their relationships to diseases and other conditions.

## 3 Results

Of the 18 tissue samples, 15 (83.3%) were from females, aged 51 to 82 years. The lesions primarily affected the bilateral buccal mucosa (88.9%) followed by the attached gingiva (11.1%). The characteristics of the samples are presented in Table 1. After sequencing, it was observed that eight samples (44.4%) had a TMB > 0, while 10 (55.6%) had a TMB of zero (Table 1). The TMB values showed a nonnormal distribution; therefore, group comparisons were performed using the Mann–Whitney *U*‐test (*α* = 0.05) with EasyMedStat (version 3.35.1). Although a higher proportion of erosive OLP samples (5/9) showed a TMB greater than 0 compared to reticular OLP samples (3/9), no significant difference in TMB was found between the two clinical forms of OLP. No difference was also observed between the two groups, even excluding the two samples collected from gingiva.

**TABLE 1:** Results of tumour mutational burden (TMB) values of oral lichen planus samples included in the study.

Sample	Predominant clinical presentation	Age	Sex	Sample location^a^	Other sites affected	Symptomatic	TMB
1	Erosive	53	F	Buccal mucosa	Gingiva	Yes	0
2	Erosive	59	F	Buccal mucosa	Gingiva	Yes	0.88
3	Erosive	57	F	Buccal mucosa	Gingiva	Yes	0.87
4	Erosive	54	F	Gingiva	—	Yes	0
5	Erosive	60	F	Buccal mucosa	Lower and inferior lip	Yes	1.76
6	Erosive	51	F	Buccal mucosa	—	Yes	0
7	Erosive	57	F	Buccal mucosa	Tongue	Yes	0
8	Erosive	58	F	Buccal mucosa	Tongue	Yes	1.83
9	Erosive	62	F	Buccal mucosa	Lower lip and tongue	No	0
10	Erosive	69	F	Buccal mucosa	—	Yes	0.87
11	Reticular	66	F	Buccal mucosa	—	Yes	1.78
12	Reticular	82	M	Buccal mucosa	—	Yes	0.87
13	Reticular	58	F	Buccal mucosa	—	Yes	0
14	Reticular	73	M	Buccal mucosa	Tongue	Yes	0
15	Reticular	52	M	Buccal mucosa	Tongue	No	0
16	Reticular	59	F	Buccal mucosa	Tongue	No	0
17	Reticular	37	F	Buccal mucosa	Tongue	No	0
18	Reticular	67	F	Gingiva	—	No	4.65

^a^ Sample collection site. All cases with lesions in the buccal mucosa had bilateral manifestations.

Somatic mutations were identified in four samples from each group: reticular and erosive lesions. Gene variants were detected in the following genes: *KDM5A, PALB2, PTPRD, ATRX, CD274 (PD‐L1), SMC3, EZH2, ETV6, BRCA2, ALPK2* and *PSMA6*. Descriptions of the variants and their clinical significance interpretations are in Table 2.

**TABLE 2:** Somatic variants found in the included oral lichen planus samples.

Sample	Clinical presentation	Gene	Variant	Variant interpretation	ClinVar accession
2	Erosive	*KDM5A* (epigenetic)	Heterozygous c.3568t>c	Not reported	
3	Erosive	*PALB2* (tumour, suppressor, DNA damage and repair)	Heterozygous c.575c>t	Not reported	
5	Erosive	*PTPRD* (cell cycle and immune response)	Heterozygous c.5369c>g	Not reported	
		*ATRX* (tumour suppressor)	Heterozygous c.2382c>g	Not reported	
8	Erosive	*CD274* (immune response)	Heterozygous c.39g>t	Not reported	
		*SMC3* (cell cycle, DNA damage and repair)	Heterozygous c.2164g>a	Not reported	
10	Reticular	*EZH2* (epigenetics)	Heterozygous c.71g>a	Not reported	
11	Reticular	*ETV6* (transcription factor)	Heterozygous c.202t>c	Missense—uncertain significance	ClinVar; [VCV002976963.1]
		*BRCA2* (tumour suppressor, DNA damage and repair)	Heterozygous c.1357c>g	Missense—uncertain significance/Likely benign	ClinVar; [VCV000629359.12]
12	Reticular	*ALPK2* (cancer‐related gene)	Heterozygous c.116c>g	Missense—uncertain significance	ClinVar; [VCV001739134.2]
18	Reticular	*PSMA6* (antigen presentation)	Heterozygous c.221t>c	Not reported	

## 4 Discussion

The TMB is associated with the progression of tumour malignancy and reflects the oncogenic potential of various tumours (Ladányi and Tímár 2020; Chalmers et al. 2017). The accumulation of somatic mutations in normal or potentially malignant cells is crucial for understanding the evolution of cancer.

Some studies have examined the TMB in potentially malignant lesions, showing that this marker gradually increases as the disease suffers transitions from a benign to a malignant neoplasm (Hu et al. 2021; Li et al. 2021). Progressive increase of tumour mutation burden from hyperplasia, to dysplasia, and to invasive oral squamous cell carcinoma is reported in the literature (William et al. 2019). An additional study found a mean TMB of 1.1 mutations/Mb in oral leukoplakia, with values increasing, particularly in patients progressing from oral leukoplakia to oral squamous cell carcinoma (Hanna et al. 2024). A large integrative analysis of genomic and transcriptomic data of normal, tumour and co‐occurring leukoplakia tissues from patients with gingivobuccal cancer found a significantly higher somatic mutation rate per Mb in leukoplakia in patients with oral tumours compared to those without it (Ghosh et al. 2022). Interestingly, oral dysplasia surrounding oral squamous cell carcinoma also shows increased TMB (Jensen et al. 2024).

Our data showed that all samples had low TMB values (≤ 10 mutations/Mb) with no differentiation between the erosive and reticular variants, suggesting that genetic mutations occur similarly in both variants. However, a higher proportion of erosive OLP samples (5/9) showed a TMB greater than 0 compared to reticular OLP planus samples (3/9). While this finding should be interpreted with caution due to the limited sample size, it may align with previous reports suggesting a potentially higher malignant transformation risk in the erosive subtype.

Several factors have been consistently reported in the literature regarding the genetic factors involved in malignant transformation, particularly aneuploidy and loss of heterozygosity, both of which can lead to chromosomal instability and thus influence malignant transformation. Chromosomal and genomic instabilities are crucial for dysplastic processes and carcinogenesis (Alaizari et al. 2018). Aneuploidy is associated with a 3.12‐fold increased risk of malignant transformation of oral dysplasia (Castagnola et al. 2015). In OLP, a high rate of aneuploidy is associated with an elevated risk of malignant transformation (Yahalom et al. 2016). Loss of heterozygosity is associated with progression of oral dysplasia to oral squamous cell carcinoma, making it a potential predictive factor in oral dysplasia (Odell 2021). It is important to note that the TMB is not necessarily related to loss of heterozygosity.

The somatic mutations in the *PTPRD*, *PSMA6 genes* and particularly the *CD274* (*PDL1*) gene, identified in the samples, are associated with immune response and inflammation regulation. In contrast, *PALB2*, *ATRX* and *BRCA2* genes are linked to tumour suppression, as they are heavily involved in DNA repair. Mutations in the *PALB2* and *BRCA2* genes are commonly associated with breast, ovarian and pancreatic cancers. However, mutations in these genes can also lead to the accumulation of mutations in other cancer types, including squamous cell carcinoma (Borkowska et al. 2021). Another study reported that patients with oral leukoplakia that progressed to oral squamous cell carcinoma might have had an increased frequency of exomic variants, particularly in genes involved in the DNA damage repair pathway, such as the *BRCA* gene (Farah et al. 2019). Previous studies have also demonstrated that lesions progressing to oral squamous cell carcinoma commonly have mutations in DNA repair genes and apoptotic pathways, primarily *TP53*, *CELSR1* and *CASP8* (Xie et al. 2022). Also, since all the genetic variants in the cohort appeared in the sample from only one patient, no genetic drivers could be identified. Future studies with broader cohorts, including samples from patients with dysplasia, will enable further exploration of the relationships between OLP, somatic mutations and the TMB.

The lower risk of malignant transformation in OLP compared with that in oral leukoplakia might also account for the lower TMB values. It is important to consider that the small sample size may have limited the ability to detect subtle differences in TMB between OLP variants. Furthermore, as the analysed samples were fixed in formalin and embedded in paraffin wax, DNA degradation during tissue processing may have impaired determination of the TMB in some samples. In addition to the limited sample size, the absence of follow‐up data regarding patient outcomes represents another relevant limitation. On the other hand, this pilot study is innovative and may serve as a reference for future research exploring TMB in erosive and reticular lichen planus cohorts across different countries.

In conclusion, erosive and reticular OLP had a low TMB. Although these data show that genetic mutational events occur similarly in these two clinical variants, further molecular studies are necessary to confirm these findings.

## Author Contributions

**Priscila Laiza Rubim Leão:** conceptualization, writing – review and editing, methodology, investigation, writing – original draft. **Rennan Garcias Moreira:** conceptualization, methodology, data curation, writing – review and editing, investigation. **Fernanda Faria Rocha:** investigation, methodology, writing – review and editing. **Laura de Freitas Xavier:** methodology, writing – review and editing. **Larissa Rany Martins‐Chaves:** investigation, writing – review and editing. **Ana Carolina Carneiro Batista de Oliveira:** investigation, writing – review and editing. **Soraya de Mattos Camargo Grossmann Almeida:** investigation, writing – review and editing. **Silvia Ferreira de Sousa:** investigation, writing – review and editing. **Marina Gonçalvez Diniz:** investigation, writing – review and editing. **Roberta Rayra Martins‐Chaves:** investigation, methodology, conceptualization, writing – review and editing. **Ricardo Santiago Gomez:** supervision, project administration, funding acquisition, writing – original draft, writing – review and editing, conceptualization, methodology, investigation.

## Conflicts of Interest

The authors declare no conflicts of interest.

## Data Availability

The authors have nothing to report.
